# First global status report on drowning prevention identifies opportunities to scale up efforts

**DOI:** 10.1136/ip-2025-045821

**Published:** 2025-07-23

**Authors:** Amy E Peden

**Affiliations:** 1 School of Population Health, University of New South Wales, Sydney, New South Wales, Australia; 2 Discipline of Public Health and Tropical Medicine, College of Public Health, Medical and Veterinary Sciences, James Cook University, Townsville, Queensland, Australia

**Keywords:** Advocacy, Policy, Drowning, Natural disaster, Recreation / Sports

 Although Friday the 13th may be viewed as unlucky for some, Friday the 13th of December 2024 was an historic moment for global drowning prevention efforts. It saw the release of the first Global Status Report on Drowning Prevention by the WHO, documenting progress in preventing drowning across 139 member states.^1^

As a preventable cause of death and injury, drowning is estimated to have claimed the lives of 300 000 people in 2021.^2^ Males, residents of low-income countries, and children and young people are over-represented in the data (table 1). Beyond demographic factors such as age and sex, a range of additional risk factors for drowning are discussed in the report, including occupational exposure, climate-related risks including flooding and heatwaves, and migration and refuge-seeking among others. Information on location and activity prior to drowning can inform prevention efforts, with respondents to the report highlighting rivers and lakes and recreational activities as key concerns.^1^

**Table 1:** Estimated counts, proportion of global drowning burden and rates of drowning deaths per 100 000 population by country income level, 2021

	Number of drowning deaths	Percentage of global drowning burden	Rate per 100 000 population
Low-income countries	39 000	13%	5.8
Lower-middle income countries	147 000	49%	4.9
Upper-middle income countries	89 000	30%	3.2
High-income countries	24 000	8%	1.7

Source: Global Health Estimates. Available online: https://www.who.int/data/global-health-estimates (date cited: 29-06-2025).

Great strides have been made in preventing drowning, with the report identifying a 38% reduction in drowning rates globally since the year 2000.^1^ However, we should be cautious in our optimism. Current estimates of fatal drowning burden exclude drowning deaths due to water transport (such as shipping and ferry disasters and boat capsize) and environmental disasters (think floods, storm surges and tsunami). This exclusion almost halves drowning figures reported in high-income contexts like Australia,^3^ and in low- and middle-income countries more prone to these events, the undercount will be even greater.

Improving data estimates would appear to be a priority to ensure appropriate resourcing and attention among policy makers and donors. The Global Status Report showcased member states’ own data on fatal drowning burden, in addition to data derived from WHO’s Global Health Estimates (GHE).^1^ Countries’ own estimates of drowning burden were both higher and lower than GHE-reported data, with much variation between the two, suggesting a need for commitment to accurately recording drowning events.

Beyond the epidemiology of drowning, the Global Status Report on Drowning Prevention examines member states’ policy and legislative approaches to preventing drowning, as well as the extent to which community-based risk reduction measures have been implemented^1^ (table 2). From a legislative perspective, the report highlights that there is much that can be done to strengthen such approaches. Just 24% of member states reported that drowning prevention is addressed through national disaster risk management policy. This is despite drowning being the leading cause of death during times of flood.^4^ Furthermore, given alcohol is a significant risk factor for drowning,^5^ it is concerning that just 26% of countries have national legislation to regulate alcohol use/sale around public water bodies.

**Table 2:** National and subnational coverage of selected legislative and policy and community-based approaches for preventing drowning (n=139)

	Percentage of participating countries with national coverage	Percentage of participating countries with national and subnational coverage	Percentage of participating countries with subnational coverage	Percentage of participating countries with no coverage or did not know
Legislation and policy				
National disaster risk management policies	24%	3%	4%	69%
Legislation for safety of passenger water-transport vessels	78%	1%	2%	19%
National legislation mandating lifejacket use	65%	–	17%	18%
National legislation for swimming pool fencing	9% (public pools) 1% (private pools) 4% (both)	–	–	86%
Alcohol-regulations	26%	–	24%	50%

	Percentage of participating countries implementing at national level	Percentage of participating countries with subnational implementation with extensive reach	Percentage of participating countries with subnational implementation with limited reach	Percentage of participating countries where approach is not implemented or did not know
Community-based approaches				
Installation of barriers near water	20%	6%	40%	34%
Swimming and water safety training into national school curricula	22%	4%	32%	42%
Day care services for pre-school children	28%	7%	27%	37%
Search and rescue services	73%	12%	9%	5%
Lifeguard services	38%	15%	30%	16%
First responder training	33%	11%	34%	21%
Disaster warning systems	71%	11%	6%	11%
Freely available weather alerts	81%	10%	4%	5%
Building community disaster resilience	42%	13%	29%	16%
Community flood risk mitigation	73%	9%	10%	8%

Extensive reach was used when an intervention was only implemented in certain states, provinces or districts in a country, but in these areas the intervention has coverage such that all people who are able to benefit from the intervention have access to it. Limited reach means the intervention is only implemented in certain states/provinces/districts in the country, and in these areas, implementation is patchy and not all people have access to it.^1^

More positively, 79% of participating countries reported at least one national law setting minimum requirements for the seaworthiness and operation of domestic passenger vessels,^1^ though improvements could be made regarding vessel safety inspections (44% of countries), requiring lifesaving equipment on board (38% of countries) and emergency plans (12% of countries). Evidence supports the effectiveness of mandatory wear lifejacket legislation^6^ and pleasingly, 65% of participating countries report having national legislation mandating lifejacket use during recreational boating and/or on passenger transport vessels.

Despite the proven effectiveness of four-sided isolation pool fencing as a drowning prevention measure,^7^ including contributing to a 63% reduction in drowning rates among children 0–4 years in Australia,^8^ 86% of countries report having no laws for fencing around either public or private swimming pools. This represents an opportunity to strengthen legislation alongside enforcement measures and education.

The report also documents implementation of a range of drowning prevention interventions, known to be evidence-based, cost-effective, scalable and adaptable to different contexts.^9^ Table 2 reports the extent of national and subnational coverage of these, including the proportion of subnational coverage with extensive and limited reach. Community-based approaches such as freely available weather alerts (81% national coverage), search and rescue services and community flood risk mitigation (73% national coverage respectively) appear to have good coverage, with regional variation.^1^ Technological advances may further support innovation and population-level coverage of these initiatives into the future.^10^

Conversely, there are opportunities to strengthen national uptake of installation of barriers near water (20% national coverage), incorporation of swimming and water safety training into school curricula (22% national coverage) and daycare services for pre-school children (28% national coverage).^1^ Given that the lowest national coverage exists for interventions known to reduce drowning risk most effectively for children and young people under 15 years of age, a cohort which accounts for 43% of unintentional drowning fatalities,^1^ there is an urgent need to expand national implementation.

With the uptake of recommended interventions, it is clear that lives will be saved. Further economic analysis of the costs of drowning and the relative saving of implementing effective interventions^11 12^ will be a vital part of increasing intervention coverage over the next decade. Though the multisectoral nature of drowning can make gaining political traction challenging,^13^ with the Global Status Report indicating just 40% of participating countries have a national coordination mechanism for drowning prevention,^1^ it also provides opportunity.

As a field of research and practice, drowning prevention is expanding rapidly^14^ and the Global Status Report on Drowning Prevention highlights many opportunities to enhance collective efforts. This may be adding to the evidence base through intervention evaluation or supporting country efforts to develop national drowning prevention strategies, which only 26% of participating countries have.^1^ It may be fostering research across regions and countries where drowning burden is greatest,^15^ or ensuring migrants and other underserved populations are supported to reduce their drowning risk.^16^

Researchers and practitioners can also play an important role in public awareness and advocacy on drowning, communicating how drowning happens and how to prevent it. On World Drowning Prevention Day (July 25th),^17^ I encourage everyone to reflect on their role in preventing drowning, be it enrolling yourself or your children in swimming lessons, talking about drowning prevention to others or just enjoying the water safely. The theme of World Drowning Prevention Day 2025 is ‘Your Story Can Save A Life’. Let’s all think about what story we will write to enhance water safety for ourselves, our loved ones and our community.
